# Supplementary material for: Deterministic quantum dot cavity placement using hyperspectral imaging with high spatial accuracy and precision
Source: Nano Converg. 2025 Jul 16;12:36. doi: 10.1186/s40580-025-00501-5 (PMC12267725; doi:10.1186/s40580-025-00501-5)
Supplement: Supplementary file 1 — Supplementary Material 1 [file 40580_2025_501_MOESM1_ESM.docx]

Supplementary information

**Deterministic Quantum Dot Cavity Placement Using Hyperspectral Imaging with High Spatial Accuracy and Precision.**

Quirin Buchinger^1, *^, Constantin Krause^1^, Aileen Zhang^1,2^, Giora Peniakov^1^, Mohamed Helal^1^, Yorick Reum^1^, Andreas Theo Pfenning^1^, Sven Höfling^1^, and Tobias Huber-Loyola^1^

^1^ Julius-Maximilians-Universität Würzburg, Physikalisches Institut, Lehrstuhl für Technische Physik, Am Hubland, 97074 Würzburg, Deutschland

^2^ University of Arizona, Wyant College of Optical Sciences, 1630 E University Blvd, Tucson Arizona, USA

* Corresponding author: Quirin Buchinger

E-mail address: quirin.buchinger@uni-wuerzburg.de

**S1: Displacement-polarization relation for spectrally detuned emission**


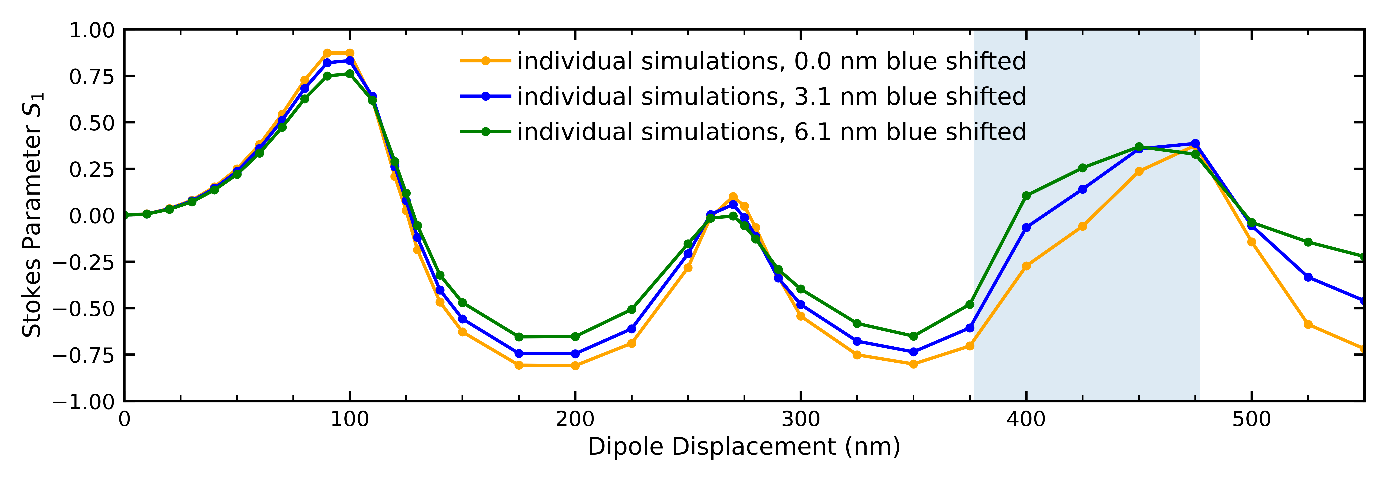


Fig. S1: Relation between the displacement of an emitter inside a circular Bragg grating (CBG) and the polarization of the emitted light. The yellow curve was previously reported in Ref. [1]. A spectral shift of the dipole emission and the cavity wavelength only slightly modifies the relationship between displacement and emitted polarization, especially for small displacements. The cavity mode linewidth of the simulated CBG is 6.7 nm FWHM.

**S2: Intentionally displaced CBGs**

We intentionally displaced 13 (8) CBGs by 95 nm in x- (y-) direction. During measurement, the lab-frame polarization basis was aligned such that the H and V axes coincided with the direction of displacement. As expected, we observe a positive (negative) S_1_ for the CBGs displaced in x- (y-) direction (Fig. S2 a). A few outliers are observed in both sets. These may result from fabrication deviations or from the fact that the intended displacement is close to the first maximum in Fig. S1. Including the outliers we find mean values of S_1,x,raw_= (0.34 ± 0.43) and S_1,y,raw_= (-0.64 ± 0.22), indicating significant linear polarization. When excluding the outliers from the dataset we find S_1,x,corr_= (0.61 ± 0.09) and S_1,y,corr_= (-0.75 ± 0.08). Compared to the non-displaced devices, S_2_ is higher, while the mean value of S_3_ is similar but exhibits a higher spread (Fig. S2 b-c). The tendency towards negative S_2_ may arise from small additional shift perpendicular to the displacement direction or from a slight misalignment between the displacement direction and polarization basis. The linear degree of polarization for the devices is Π_x,raw_ = (0.58 ± 0.15) and Π_y,raw_ = (0.69 ± 0.20), respectively when analyzing the whole dataset and Π_x,corr_ = (0.66 ± 0.08) and Π_y,corr_ = (0.79 ± 0.08), when excluding the outliers. Using the relation shown in Fig. S1 and published in Ref. [1], we extract displacement. However, as the indented 95 nm displacement lies near the first maximum in the displacement-polarization relation, the extracted displacement is ambiguous: for example, a linear degree of polarization of Π = 0.80 could correspond to either ~84 nm or ~104 nm displacement. Lacking further information, we always assign the lower value. Most likely, this is not always the case. The resulting displacements are $\text{x}_{\text{displacement,raw}}=(71.8 \pm9.3) \text{nm}$ and $\text{y}_{\text{displacement,raw}}=(78.4 \pm12.3) \text{nm}$, respectively, when analyzing the whole dataset and $\text{x}_{\text{displacement,corr}}=(76.3 \pm4.3) \text{nm}$ and $\text{y}_{\text{displacement,corr}}=(84.4 \pm5.8) \text{nm}$. The spread is comparable to that observed from non-displaced devices (see main text, Section 3.3). The absolute value is likely an artefact, due to the ambiguity in the polarization-displacement relation and the fact that we arbitrarily chose the smaller value of displacement.


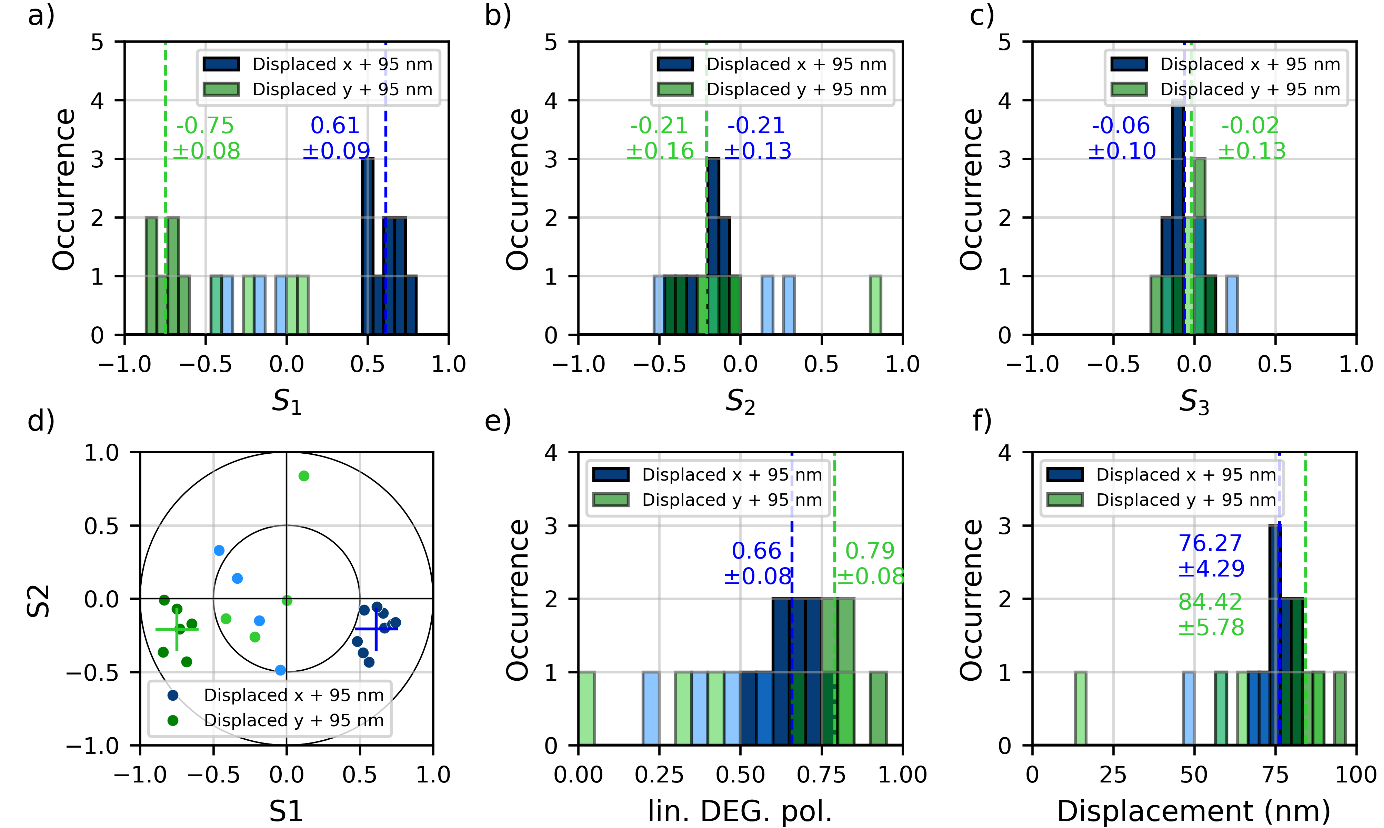


Fig. S2: a)-d) Stokes parameter for CBGs intentionally displaced by 95 nm in the x- and y-direction. e) Linear degree of polarization, calculated from S_1_ and S_2_. f) Displacement values translated from the linear degree of polarization using the relation from Fig. S1. Note that these results should be interpreted with caution, as the polarization-displacement relation is not unique near the intended 95 nm displacement. Outliers are shown in lighter colors and excluded from the mean value calculations.

**S3: Lifetime measurements**

To demonstrate the potential Purcell enhancement in our deterministically placed devices, we present a time-resolved photoluminescence measurement of a single exciton line from a QD coupled to a placed CBG on the investigated sample. A biexponential fit convoluted with the system response function reveals a lifetime of approximately 47 ps indicating a Purcell factor around 20 (Fig. S3 a). The spectrum of the QD is shown in (Fig. S3 b). The measured line is labeled and was identified to be the neutral exciton using cross-correlation measurements.


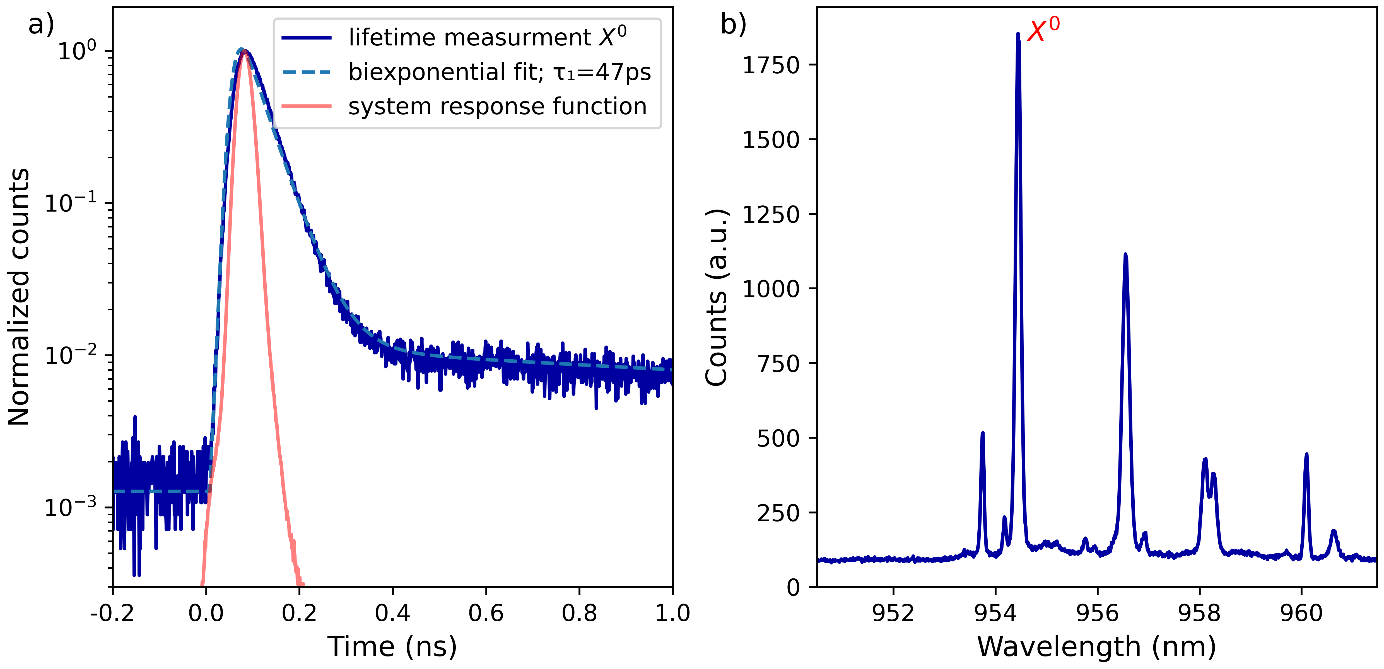


Fig. S3: a) Lifetime measurement of the exciton line labeled in b).

**References**

1. G. Peniakov, Q. Buchinger, M. Helal, S. Betzold, Y. Reum, M.B. Rota, G. Ronco, M. Beccaceci, T.M. Krieger, S.F.C. Da Silva, A. Rastelli, R. Trotta, A. Pfenning, S. Höfling, T. Huber‐Loyola, Laser & Photonics Reviews (2024) doi:10.1002/lpor.202300835
